# Development and evaluation of a colorectal cancer screening method using machine learning‐based gut microbiota analysis

**DOI:** 10.1002/cam4.4671

**Published:** 2022-03-22

**Authors:** Yusuke Konishi, Shintaro Okumura, Tomonori Matsumoto, Yoshiro Itatani, Tsuyoshi Nishiyama, Yuki Okazaki, Masatsune Shibutani, Naoko Ohtani, Hisashi Nagahara, Kazutaka Obama, Masaichi Ohira, Yoshiharu Sakai, Satoshi Nagayama, Eiji Hara

**Affiliations:** ^1^ Research Institute for Microbial Diseases (RIMD) Osaka University Suita Japan; ^2^ The Cancer Institute Japanese Foundation for Cancer Research (JFCR) Tokyo Japan; ^3^ Graduate School of Medicine Kyoto University Kyoto Japan; ^4^ Osaka City University Graduate School of Medicine Osaka Japan; ^5^ The Cancer Institute Hospital, JFCR Tokyo Japan; ^6^ Uji‐Tokushukai Medical Center Uji Japan; ^7^ Immunology Frontier Research Centre (IFReC) Osaka University Suita Japan; ^8^ Center for Infectious Disease Education and Research (CiDER) Osaka University Suita Japan

**Keywords:** biomarkers, colorectal cancer, next generation sequencing, screening

## Abstract

Accumulating evidence indicates that alterations of gut microbiota are associated with colorectal cancer (CRC). Therefore, the use of gut microbiota for the diagnosis of CRC has received attention. Recently, several studies have been conducted to detect the differences in the gut microbiota between healthy individuals and CRC patients using machine learning‐based gut bacterial DNA meta‐sequencing analysis, and to use this information for the development of CRC diagnostic model. However, to date, most studies had small sample sizes and/or only cross‐validated using the training dataset that was used to create the diagnostic model, rather than validated using an independent test dataset. Since machine learning‐based diagnostic models cause overfitting if the sample size is small and/or an independent test dataset is not used for validation, the reliability of these diagnostic models needs to be interpreted with caution. To circumvent these problems, here we have established a new machine learning‐based CRC diagnostic model using the gut microbiota as an indicator. Validation using independent test datasets showed that the true positive rate of our CRC diagnostic model increased substantially as CRC progressed from Stage I to more than 60% for CRC patients more advanced than Stage II when the false positive rate was set around 8%. Moreover, there was no statistically significant difference in the true positive rate between samples collected in different cities or in any part of the colorectum. These results reveal the possibility of the practical application of gut microbiota‐based CRC screening tests.

## Graphical abstract

We have established a new machine learning‐based colorectal cancer (CRC) diagnostic model using the gut microbiota as an indicator. Validation using independent test datasets showed that the true positive rate of our CRC diagnostic model increased substantially as CRC progressed from stage I to stage II. These results reveal the possibility of the practical application of gut microbiota‐based CRC screening tests.

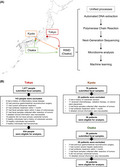

## 1 INTRODUCTION

In developed countries, the morbidity and mortality of colorectal cancer (CRC) are increasing year by year, and its countermeasures are becoming an urgent issue.^1^ Since effective therapeutic drugs for CRC are still under development, the only effective measure at the moment is early detection and surgical removal of CRC. Thus, there is a need for a simple and accurate CRC screening test. Currently, the most widespread screening tests for CRC are colonoscopy and the fecal occult blood test (FOBT).^1^ Although the colonoscopic examination achieves an accurate and sensitive diagnostic test for CRC detection, it is invasive, expensive, *labor‐intensive,* and time consuming. Thus, it is difficult to use it for a population‐wide CRC screening.^1^ On the other hand, FOBT is a non‐invasive, simple, and inexpensive screening test for CRC detection, but often gives false positive results especially when applied to those with bleeding by hemorrhoids or menstruation. Furthermore, the sensitivity of FOBT for early‐stage CRC and proximal colon cancer is not adequately high because of the difficulty to detect the low amount of blood in the stool.^2^ Therefore, there is a need to develop a new non‐invasive, simple, and effective CRC screening test that can compensate for the problems of the FOBT.

The gut microbiota is an ecosystem created by a wide variety of bacteria that reside in the intestinal lumen. It is known that the gut microbiota helps to maintain the homeostasis of the organism through the construction of the host's immune system and assistance in food digestion.^3^, ^4^, ^5^ However, when the composition of the gut microbiota is disrupted by overeating, an imbalanced diet, or antibiotic medication, the number of useful bacteria decreases, and pathogenic bacteria proliferate instead, causing intestinal and systemic inflammation, and metabolic disorders.^3^, ^4^, ^5^ Accumulating evidence indicates that gut bacteria are also involved in tumorigenesis in the liver^6^, ^7^ and colon.^8^, ^9^, ^10^ Notably, *Fusobacterium nucleatum,* ^11^, ^12^, ^13^ enterotoxigenic *Bacteroides fragilis,* ^14^ *pks* ^*+*^ *Escherichia coli,* ^15^, ^16^, ^17^, ^18^ and *Peptostreptococcus anaerobius* ^19^ are reportedly involved in the development of CRC. Furthermore, we have recently reported that *Porphytomonas gingivalis* and *Porphytomonas asaccharolytica* may promote the development of CRC through the production of butyrate.^20^ Thus, examination of gut microbiota to detect those pathogenic bacteria would be a promising method to screen CRCs. However, since these pathogenic bacteria are not detected in all patients with CRCs and some of these bacteria are also detected in healthy individuals, screening for CRC cannot be satisfactorily performed if the presence of these pathogenic bacteria alone is used as markers.^13^, ^21^, ^22^

In recent years, several studies have been conducted to detect the differences in the gut microbiota between healthy individuals and CRC patients using machine learning‐based meta‐sequencing analysis of bacterial DNA, and to use this information for the diagnosis of CRC.^23^, ^24^, ^25^, ^26^, ^27^ However, most of the studies have small sample sizes, and some of them do not have independent test datasets, but only cross‐validate the diagnostic model using the training dataset used to create the model.^23^, ^24^, ^25^, ^26^, ^27^ Since machine learning‐based diagnostic models can cause overfitting if the sample size is small.^25^ Furthermore, cross‐validation is not an appropriate validation method because the results can reflect the characteristics of the training data and cause an overfitting problem. Therefore, it is essential to develop a diagnostic model using a training dataset with sufficient sample size, and at the same time, to validate it using an independent test dataset. Furthermore, some studies have used sequence data published in databases to increase the sample size. However, since differences in experimental conditions and sample population characteristics may affect the prediction results of machine learning models, care should be taken in interpreting the results obtained. It should also be noted that although shotgun metagenomic sequencing analysis has higher bacterial classification accuracy than meta‐16S rRNA gene sequencing analysis, it is currently difficult to use for primary screening of large populations because of the high cost of analysis and the need for high‐performance computers to analyze large amounts of data.

To solve the above problems, in this study, we attempted to develop a new diagnostic model for CRC screening test using meta‐sequencing analysis of gut bacterial 16S rRNA genes with a carefully designed machine learning approach. Notably, our model has been created using a sufficient number of training datasets and validated using independent test datasets collected from three hospitals in different regions of Japan. With these improvements, we succeeded in developing a more reliable CRC screening method using the gut microbiota, and we report it here and discuss the potential and limitations of the method using the gut microbiota.

## 2 MATERIALS AND METHODS

### 2.1 Human fecal sample collection

Feces were collected from study participants who visited the JFCR (Tokyo) from December 2013 to March 2015 (cohort‐1), January to September 2017 (cohort‐2), and May 2019 to August 2021 (cohort‐3), and those who visited Kyoto University Hospital (Kyoto) or Osaka City University Hospital (Osaka) from May 2019 to August 2021 (cohort‐3) using a fecal sampling tool (TechnoSuruga Laboratory). All study participants underwent colonoscopy at the time of stool collection. Colorectal cancer patients (CRC patients) were defined as patients with primary malignant epithelial colorectal tumors according to the Third English Edition of the Japanese Classification of Colorectal, Appendiceal, and Anal Carcinoma.^2^ Advanced adenoma patients were defined as patients with colorectal adenomas larger than 10 mm in diameter. Healthy individuals (HI) were defined as individuals without colorectal cancers nor colorectal advanced adenomas. HI were classified into two groups: clean HI who had no colorectal adenomas and HI with colorectal polyps smaller than 10 mm in size. We excluded those with a history of inflammatory bowel disease, prior gastrointestinal reconstructive surgery, severe liver dysfunction, anticancer and/or antibiotic treatment within 1 month, stool collection within 3 days of colonoscopy, and those without access to detailed clinical information. Patients who received chemotherapy, radiation therapy, or colonic stent placement before fecal sample collection, who had fecal samples collected after endoscopic resection of tumors, or patients whose tumors were not primary colorectal tumors (e.g., squamous cell carcinoma of the anal canal cancer, metastasis or direct invasion of other cancers to the large intestine) were also excluded. HI with a history of colorectal cancers, or with malignant tumors other than colorectal cancer or abnormal endoscopic findings, such as enteritis and hamartomas at the time of stool collection were excluded. Written informed consent was obtained from all participants for the use of anonymized samples and the publication of the patients' clinical information under the protocol approved by the ethics committee of the JFCR hospital, Kyoto University Hospital, and Osaka City University Hospital. The tumor profiles of CRC patients were classified based on the Third English Edition of the Japanese Classification of Colorectal, Appendiceal, and Anal Carcinoma.^2^

### 2.2 16S rRNA gene sequencing analysis and microbiome analysis

Bacterial DNA extraction from fecal samples was performed using a QIAamp Fast DNA Stool Mini Kit (QIAGEN) (samples of cohort‐1) or a Magtration System 12GC (Precision System Science) in TechnoSuruga Laboratory (samples of cohort‐2) or a GENE STAR PI‐480 automated DNA isolation system (Kurabo Industries, Ltd., Osaka, Japan) (samples of cohort‐3). The polymerase chain reaction (PCR) amplification of the V1‐V2 region of the bacterial 16S rRNA gene was performed using KAPA HiFi Hot Start Ready Mix (Roche) with universal 16S rRNA primers followed by the secondary amplification adding the Illumina flow cell adapters and indices. The PCR primers used are shown in Table S1. Meta‐16S rRNA gene sequencings were carried out per 192 samples on the Illumina MiSeq platform (Illumina Inc.) using MiSeq Reagent Kit v2 (Illumina Inc.) (paired‐end, 250 cycles × 2). These processes were performed at Biken Biomics, Inc. Sequencing reads were processed according to the QIIME2 (version 2020.8) pipeline.^28^ Fastq files were de‐noised with the DADA2 plugin^29^ and amplicon sequence variants (ASVs) were counted. These processes were performed separately for samples from the training data and the test data. Subsequently, de novo clustering was performed on ASVs using the VSEARCH plugin^30^ to obtain operational taxonomic units (OTUs) with a similarity of more than 99%. Open‐reference clustering based on OTUs detected from the training data were performed on the test data. Finally, the OTU counts were converted to relative abundance per sample. A phylogenetic tree was generated from the ASVs, and beta diversity analyses (principal coordinate analyses of weighted UniFrac distance) were performed with a sampling depth of 10,000 reads. Phylogenetic classification of the detected OTUs was performed by a Naive Bayes classifier trained on the SILVA 16S rRNA sequence database (version 138)^31^ in the QIIME2 pipeline. Identification of the specific bacterial species corresponding to each OTU was performed by using the 16S rRNA database provided by the National Center for Biotechnology Information (NCBI) (last modified on 12 June 2021) and a similarity search with BLAST+ (version 2.9.0).^32^

### 2.3 Statistical modeling

For the construction of the colorectal cancer screening model, h2o.automl function with 10‐fold cross‐validations in the h2o package of R (version 3.32.0.1) (https://www.h2o.ai/) was performed. After repeating this process 10 times, the StackedEnsemble_BestOfFamily model with the highest AUC for the cross‐validation predictions was selected. The h2o package displays the feature (variable) importance scaled between 0 and 1, except for stacked ensemble learning. In this study, the scaled feature importance in a stacked ensemble model was defined as the rescaled sum of the product of the scaled importance of its constituent models within a meta‐learner and the scaled importance of each feature within each model.

### 2.4 Quantitative real‐time PCR analysis

Quantitative real‐time PCR was performed on Thermal Cycler Dice® Real‐Time System III (Takara Bio Inc.) using TB Green® Premix Ex Taq™ II (Takara Bio Inc.). Universal 16S rDNA was used as internal control, and the abundances of the bacteria were expressed as relative levels to 16S rDNA. The PCR primer sequences used are shown in Table S1.

### 2.5 Statistical analysis

Statistical analysis was performed using R (version 4.0.5). The differences in the characteristics of the samples were analyzed by the Wilcoxon rank sum test and the chi‐squared test. For the performance testing of the colorectal cancer screening model, a bootstrapping method with 10,000 resamples by the pROC package of R (version 1.16.2)^33^ was used. To compare the variables of the multiple sample groups, the pairwise method with the adjustment of *p* values by the Benjamini–Hochberg false‐discovery rate correction at 0.05 was performed. The Cochran–Armitage test for trends in proportions was used to evaluate the statistical significance of trends in positive rates across colorectal cancer progression. Statistical tests were two‐tailed and *p* < 0.05 was considered significant.

## 3 RESULTS

### 3.1 Strategies for optimizing the diagnostic model of CRC

**Figure 1:**
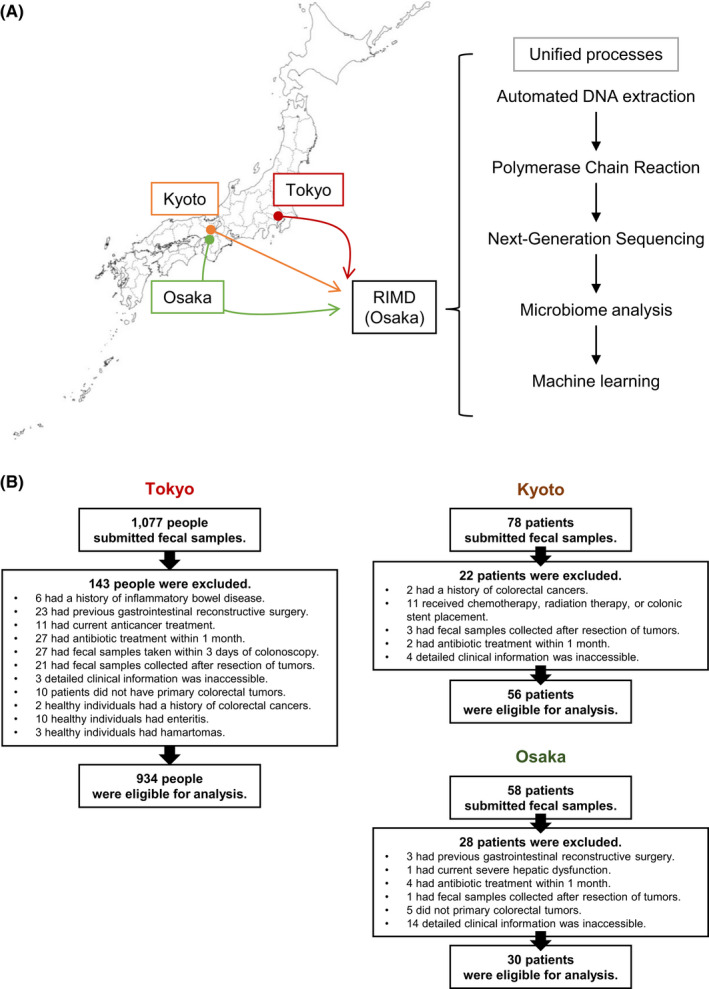
The strategy of this study. (A) flow diagram of cohort‐3. Stool samples from three hospitals in Tokyo (JFCR hospital), Kyoto (Kyoto University Hospital), and Osaka (Osaka City University Hospital) were collected in one place and analyzed using a uniform method. (B) Workflow chart for enrolling healthy individuals (HI) and CRC patients for microbiome analysis in cohort‐3

In our previous study, stool samples from healthy individuals and patients with CRC were collected twice at the JFCR hospital in Tokyo (cohort‐1 in 2013–2015 and cohort‐2 in 2017–2018) and their gut microbiota profiles were analyzed by bacterial 16S rRNA gene meta‐sequencing.^20^ As a result, we found 12 common CRC‐related bacterial species that were significantly increased in CRC patients but almost undetectable in healthy individuals in both cohorts.^20^ Unexpectedly, however, the CRC diagnostic model created by machine learning based on the dataset of cohort‐1 failed to correctly diagnose the sample of cohort‐2. To investigate the cause of this, we compared the β‐diversity of the gut microbiota between the two cohorts and found that they differed substantially (Figure S1). These two cohorts used different methods to extract DNA from stool, which may have caused differences in the gut microbiota profile and reduced diagnostic efficiency. Therefore, considering this result and the problems that have been pointed out regarding the generation of a CRC diagnostic model using the gut microbiota and machine learning,^34^, ^35^, ^36^ we attempted to develop a new CRC diagnostic model by paying attention to the following three points^1^: Create a diagnostic model for CRC screening using a sufficient number of training datasets and validate it appropriately using independent samples as test datasets.^2^ Stool collection, DNA extraction, and sequencing are performed based on a consistent pipeline to minimize data variability due to differences in operations.^3^ Test samples were collected at three hospitals located in different regions of Japan (Tokyo) to evaluate the diagnostic robustness regardless of the region of donor residency (Figure 1A).

### 3.2 Determining the sample size required to create a CRC diagnostic model

The small sample size can lead to overfitting in generating diagnostic models based on machine learning. In addition, cross‐validation, which is performed using part of the training dataset rather than being evaluated using an independent test dataset, also increases the risk of overfitting.^25^ Therefore, we attempted to estimate the number of training data samples required to create a diagnostic model for the CRC screening test using the training dataset and independent test dataset of cohort‐1. Note that because there is a slight batch effect for each sequencing run, there may be some errors when using amplicon sequence variants (ASVs), which are classified as identical only if they are 100% identical, in the analysis. To circumvent this problem, we utilized an operational taxonomic unit (OTU) clustering ASVs with 99% identity rather than ASV itself, and then create and validate a CRC diagnostic model using h2o AutoML (https://www.h2o.ai/), an open‐source machine learning platform (Figure S2). The h2o AutoML can run seven different machine learning algorithms: Generalized Linear Model, Distributed Random Forest, Extremely Randomized Trees, Gradient Boosting Machine, XGBoost, Deep learning, and Stacked Ensemble. However, it is generally known that Stacked Ensemble has the best performance, and Stacked Ensemble is further divided into Stacked Ensemble_AllModels and Stacked Ensemble_BestOfFamily (Figure S2). Although the performance of Stacked Ensemble_AllModels and Stacked Ensemble_BestOfFamily is almost the same, the model created by Stacked Ensemble_AllModels has a very large data size (Figure S2). Therefore, in this study, we decided to use Stacked Ensemble_BestOfFamily which has a manageable data size to create a CRC diagnostic model. We created a diagnostic model by varying the number of clean healthy individuals and CRC patients in the training dataset of cohort‐1, and evaluated the diagnostic efficiency of the model by calculating the AUC (area under the curve) of the ROC (receiver operating characteristic) curve using independent test dataset^37^ (Table S2 and Figure S3A). As the number of clean healthy individuals and CRC patients used to create the model was increased step by step, the value of AUC increased until the number of subjects reached 120 each (Figure S3B). However, when the sample size was increased to more than 120 subjects each, the AUC values did not increase anymore (Figure S3B), indicating that the training data of 120 clean healthy individuals and 120 CRC patients were necessary and sufficient for creating the CRC diagnostic model.

### 3.3 Development of a diagnostic model for CRC screening

Given the estimated sample size required to create a diagnostic model, samples required for the development of the CRC diagnostic model (more than 120 clean healthy individuals and CRC patients each) were newly collected in Tokyo for training data as cohort‐3. Independent samples for test data were also collected in Kyoto and Osaka in addition to Tokyo. All collected stool samples were subjected to DNA extraction, sequencing of bacterial 16S rRNA gene, and clustering of sequence data by a consistent pipeline (Figure 1A). Among a total of 1213 samples collected, 193 samples inappropriate for analysis were excluded, and 1020 samples were processed to create a CRC diagnostic model (Figure 1B). From cohort‐3, 120 samples each of clean healthy individuals and CRC patients from the Tokyo sample were selected as training data (Table 1), and a new CRC diagnostic model was generated by the Stacked Ensemble_BestOfFamily machine learning algorithm. Subsequently, the model generated from training data were evaluated by test dataset. It should be noted that we were unable to collect samples from healthy individuals in Kyoto and Osaka and that the distribution of cancer progression stage among CRC patients was uneven (Table 1). Therefore, in order to increase the number of test data, we also used the dataset of the cohort‐2, where DNA extraction from stool was performed in the same way as in the cohort‐3, and its β‐diversity was similar to that of the cohort‐3 as judged by principal coordinate analysis (PCoA) of weighted UniFrac distance^38^ (Figure S4). Thus, the test dataset was composed of all samples that were not used for training data in cohort‐3 and cohort‐2, whose specimens were processed in a consistent way (Table 1). Note that the value of AUC is highly dependent on the sample composition of the dataset. Therefore, as a measure to evaluate the diagnostic model, we decided to also compare the true positive rate (sensitivity) for each stage of cancer when the threshold of the algorithm was set to a value that is expected to result in a false positive rate (i.e., 1 ‐ specificity) of about 8%, as in Zeller et al.^26^

**Table 1:** Summary of the study participants in cohort‐3

Study population	Clean HI	HI with polyps	Advanced adenoma patients	CRC patients stage				
				0	I	II	III	IV
Training data								
Tokyo	120			22	43	31	17	7
Test data								
Tokyo	234	165	75	30	46	36	76	32
Kyoto					21	16	19	
Osaka				1	8	12	5	4
Cohort‐2 (Tokyo)	83			37	32	13	30	8

Abbreviation: HI: Healthy individuals.

**Figure 2:**
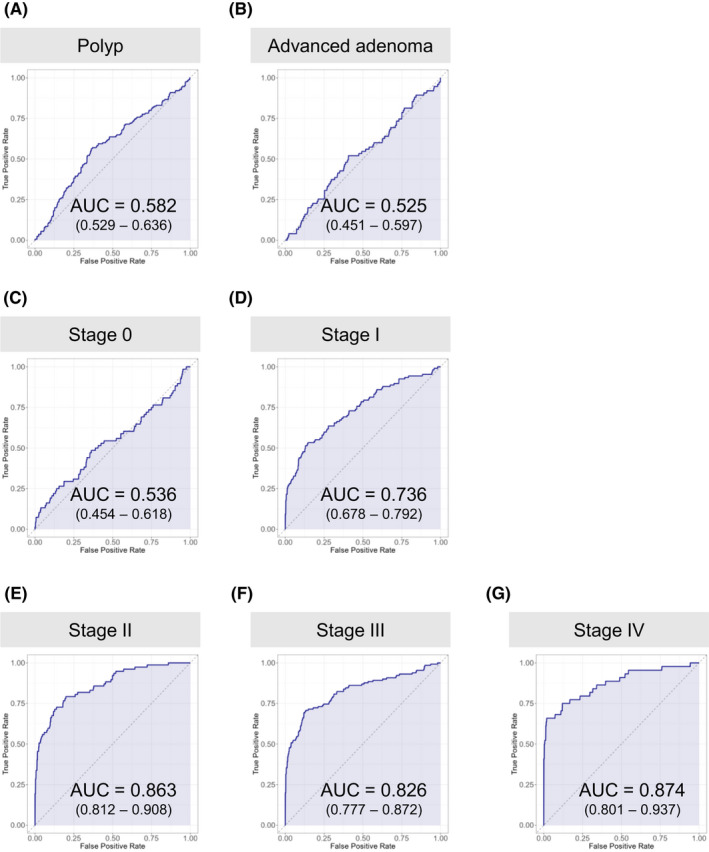
Evaluation of CRC diagnostic model by ROC curve. (A)–(G) ROC curves were drawn separately for the different sample categories and for each stage of CRC progression. Healthy individuals with colorectal polyps (A), advanced adenoma patients (B), Stage 0 (C), Stage I (D), Stage II (E), Stage III (F), Stage IV (G). AUC (area under the curve). Ranges in parentheses are 95% confidence intervals with 10,000 bootstrap replicates

**Figure 3:**
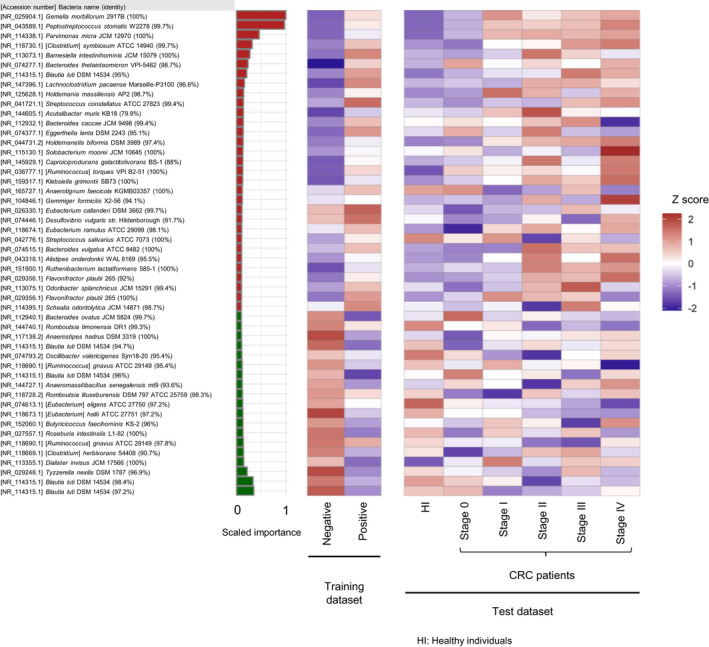
The top 50 most important bacteria (OTUs) for diagnosis in our CRC diagnostic model developed in this study. The heat map shows the mean relative abundance of the top 50 most important bacteria (OTUs) for diagnosis in our CRC diagnostic model in the training and test datasets. The bar chart shows the scaled feature (variable) importance of each OTU. The color of bar chart is red if the average relative presence ratio of positive samples is greater than the average relative presence ratio of negative samples in the training dataset, and green otherwise. The relative amounts were multiplied by 10,000 and logarithmically transformed (pseudo‐count = 1), then the mean of each sample group was calculated and finally normalized by the *z*‐score

**Figure 4:**
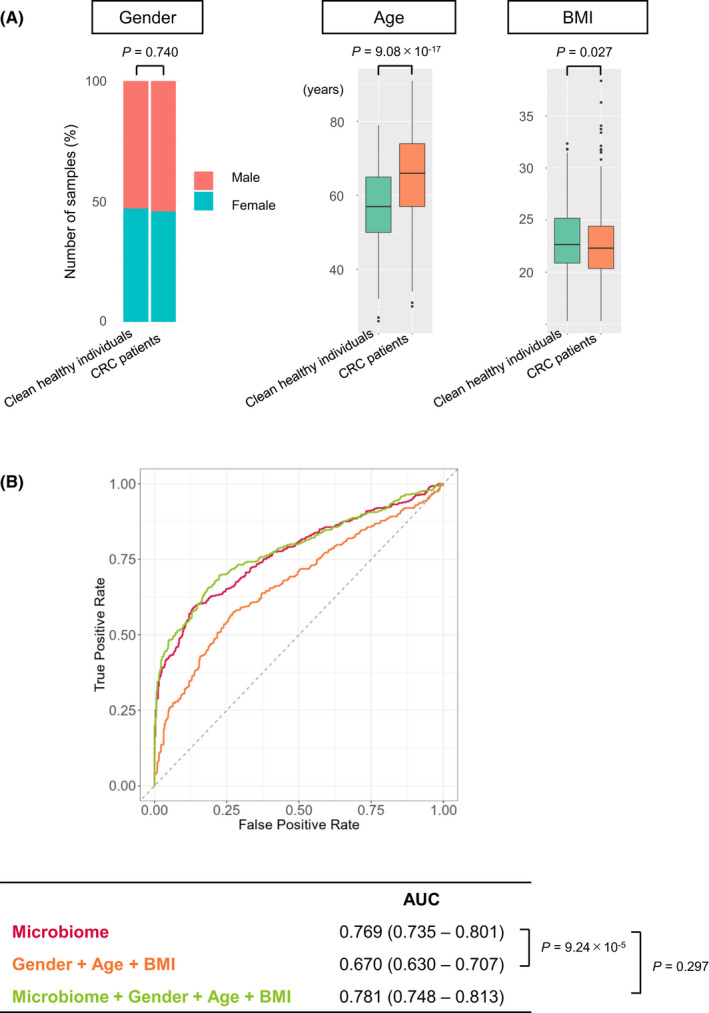
Gender, age, and body mass index (BMI) of the cohort‐3 samples. (A) Distribution of gender, age, and BMI in the test dataset (317 clean healthy individuals and 426 CRC patients). Missing data have been skipped. The boxes in the graph of age and BMI represent 25th–75th percentiles, black lines indicate the median, whiskers extend to the maximum and minimum values within 1.5× the interquartile range and dots indicate outliers. Statistical significance was determined with the chi‐squared test (gender) or the two‐tailed Wilcoxon rank sum test (age and BMI). *p* values < 0.05 were considered significant. (B) ROC curves for the test datasets of the “microbiome” model, the “Gender + Age + BMI” model, and the “Gender + Age + BMI + microbiome” model. The “microbiome” model was trained to distinguish between CRC patients and clean healthy individuals based solely on gut microbiota. The “Gender + Age + BMI” model was trained based on gender, age, and BMI. The “Gender + Age + BMI + microbiome” model was trained based on gender, age, BMI, and gut microbiome. Despite the significant differences in Age and BMI between clean healthy individuals and CRC patients (A), the AUC of the “Gender + Age + BMI” model was low. The AUC of the “Gender + Age + BMI + microbiome” model did not differ from that of the “microbiome” model. Ranges in parentheses are 95% confidence intervals with 10,000 bootstrap replicates. Statistical significance was determined with a bootstrapping method with 10,000 resamples. *p* values < 0.05 were considered significant

Judging from the independent test dataset, the AUC and true positive rate of CRC patients increased substantially as CRC progressed from stage I, with AUCs above 0.80 and true positive rates above 60% in patients with stage II or higher CRC (Figure 2 and Table 2). The true positive rate also significantly increased in correlation with the depth of tumor invasion (T classification),^2^ indicating that our model is suitable to detect advanced CRCs (Table 2). On the other hand, the true positive rate of patients with early‐stage cancers belonging to stage 0 or T0 was as low as 19.1% and the AUC = 0.536, almost indistinguishable from clean healthy individuals (Table 2 and Figure 2). Healthy individuals with colorectal polyps and patients with advanced adenoma were also indistinguishable from clean healthy individuals (Table 2 and Figure 2A and B). Consistent with these results, the mean relative abundance of the top 50 most important bacteria (OTUs) for diagnosis in our CRC diagnostic model changed after stage I of CRC (Figure 3). It is worth emphasizing that there was no significant difference in the true positive rate between the data from the three hospitals (Tokyo, Kyoto, and Osaka) (Table 3) and no statistically significant difference in the true positive rate in any part of the colorectum (Table 4). It should also be noted that although there was a statistically significant difference in age and BMI (body mass index) between the healthy individual group and the CRC patient group in the test dataset, the CRC diagnostic model obtained by training data combining gender, age, BMI, and gut microbiota composition did not significantly improve performance from the model trained on gut microbiota composition data alone (Figure 4). This result suggests that changes in gut microbiota are more strongly correlated with colorectal cancer than age or BMI.

**Table 2:** Positive rate of our CRC diagnostic model for clean healthy individuals, healthy individuals with colorectal polyps, advanced adenoma patients, and patients with CRC in different stages of CRC progression (stage and T classification)

Group	*n*	Positive rate (%)
Clean healthy individuals	317	10.7 (7.3–14.2)
Healthy individuals with polyps	165	13.3 (8.5–18.8)
Advanced adenoma patients	75	12.0 (5.3–20.0)
CRC patients		
Stage		
0	68	19.1 (10.3–29.4)
I	107	45.8 (36.4–55.1)
II	77	66.2 (55.8–76.6)
III	130	63.1 (54.6–71.5)
IV	44	68.2 (54.5–81.8)
		*p* = 7.99 × 10^–10^
T classification		
Tis	68	19.1 (10.3–29.4)
T1	77	48.1 (36.4–59.7)
T2	60	46.7 (33.3–60.0)
T3	166	66.3 (59.0–73.5)
T4	55	67.3 (54.5–80.0)
		*p* = 6.88 × 10^–11^

Ranges in parentheses are 95% confidence intervals with 10,000 bootstrap replicates. Statistical significance was determined with the Cochran–Armitage test for trends in proportions. *p* values < 0.05 were considered significant.

**Table 3:** Positive rate of our CRC diagnostic model for CRC patients in different hospitals by stage

Group	*n*	Positive rate (%)
Clean healthy individuals	317	10.7 (7.3–14.2)
CRC patients		
Stage 0/I/II		
Tokyo	112	47.3 (37.5–56.3)
Kyoto	37	59.5 (43.2–75.7)
Osaka	21	57.1 (38.1–76.2)
		*p* = 0.371
Stage III/IV		
Tokyo	108	67.6 (59.3–75.9)
Kyoto	19	68.4 (47.4–89.5)
Osaka	9	66.7 (33.3–100)
		*p* = 0.995

Ranges in parentheses are 95% confidence intervals with 10,000 bootstrap replicates. Statistical significance was determined with the chi‐squared test. *p* values < 0.05 were considered significant.

**Table 4:** Positive rate by stage of our CRC diagnostic model for CRC with a different location

Group	*n*	Positive rate (%)
Clean healthy individuals	317	10.7 (7.3–14.2)
CRC patients		
Stage 0		
Proximal colon	32	9.4 (0.0–21.9)
Distal colon	13	30.8 (7.7–53.8)
Rectum	23	26.1 (8.7–43.5)
		*p* = 0.148
Stage I		
Proximal colon	36	38.9 (22.2–55.6)
Distal colon	24	45.8 (25.0–66.7)
Rectum	47	51.1 (36.2–66.0)
		*p* = 0.544
Stage II		
Proximal colon	32	62.5 (46.9–78.1)
Distal colon	22	59.1 (36.4–77.3)
Rectum	23	78.3 (60.9–95.7)
		*p* = 0.335
Stage III/IV		
Proximal colon	45	57.8 (42.2–71.1)
Distal colon	43	58.1 (44.2–72.1)
Rectum	86	70.9 (61.6–80.2)
		*p* = 0.203

Ranges in parentheses are 95% confidence intervals with 10,000 bootstrap replicates. Statistical significance was determined with the chi‐squared test. *p* values < 0.05 were considered significant.

### 3.4 Comparison with qPCR methods for detection of specific gut bacteria

**Figure 5:**
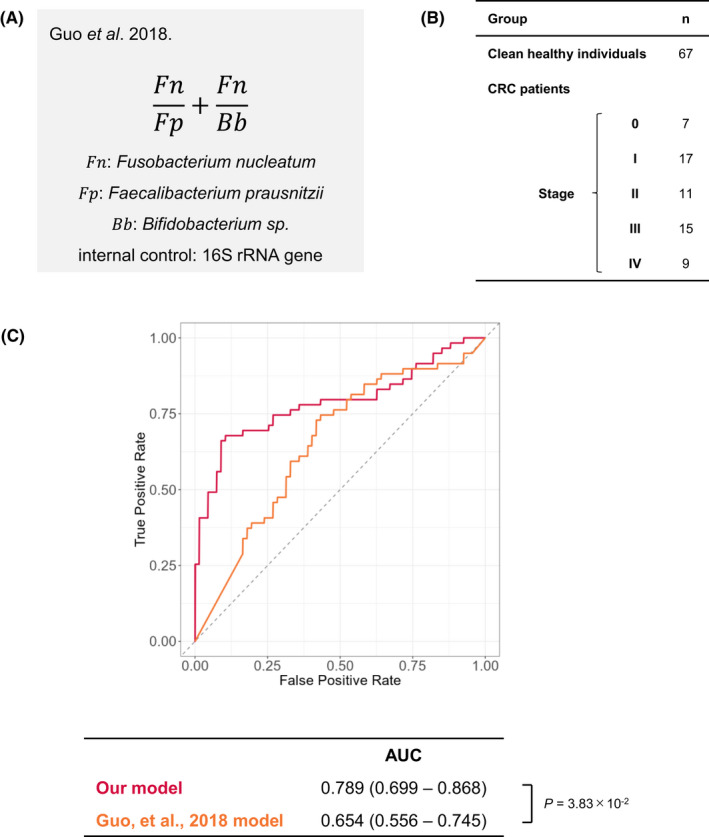
Comparison with quantitative PCR method (Guo et al., 2018). (A) Formula for Guo's model, using the relative presence of the three bacteria. (B) The sample configuration used to validate Guo's model. (C) Comparison of the ROC curves from our CRC diagnostic model and Guo's model for the samples listed in Panel B. Ranges in parentheses are 95% confidence intervals with 10,000 bootstrap replicates. Statistical significance was determined with a bootstrapping method with 10,000 resamples. *p* values < 0.05 were considered significant

Recently, Guo et al.^39^ reported that a highly accurate CRC diagnostic model was developed by combining the results of qPCR quantification of the abundance of three gut bacteria, *Fusobacterium nucleatum, Faecalibacterium prausnitzii,* and *Bifidobacterium* spp. Therefore, in order to compare the efficiency of our model and Guo's model,^39^ we randomly selected samples of 67 clean healthy individuals and 59 CRC patients from our cohort‐3, performed a qPCR analysis with the same primers as Guo et al., and applied it to Guo et al.’s diagnostic model. Although the CRC diagnostic model by Guo et al. achieved very high diagnostic accuracy with AUC = 0.964 in their dataset, their model only showed AUC = 0.654 in our cohort‐3 dataset, which was lower than our CRC diagnostic model (Figure 5). It is unclear why this difference occurred, but it may reflect differences in DNA extraction methods or regional differences between Japan and China. However, our data suggest that screening for CRC using the diagnostic model of Guo et al. would be difficult, at least in a Japanese sample.

## 4 DISCUSSION

The colon contains the highest density of metabolically active microbiota, and it is becoming clear that changes in the composition of the gut microbiota are associated with colorectal cancer.^8^, ^9^, ^10^ Although only a limited number of gut bacteria have been reported to be involved in the development of CRC,^11^, ^12^, ^13^, ^14^, ^15^, ^16^, ^17^, ^18^, ^19^, ^20^ many studies have reported increased or decreased abundance of certain gut bacteria in patients with CRC.^26^, ^27^, ^40^, ^41^ Therefore, changes in the gut microbiota may be useful for screening for colorectal cancer. In this study, we established a new machine learning‐based CRC diagnostic model using gut microbiota as an indicator, overcoming the problems that have been pointed out in the past, such as sample size and independent test datasets. However, since several attempts have already been made to use the gut microbiota as an indicator for CRC diagnosis by machine learning,^23^, ^24^, ^25^, ^26^, ^27^ it is important to compare our CRC diagnosis model with Zeller's model, which seems to be the most reliable so far because it uses independent test dataset for validation.^26^ In Zellers's model, however, the training data were small (88 healthy individuals and 53 CRC patients), and the test data included only 38 CRC patients.^26^ Furthermore, most of the healthy individual data in the test dataset were cited from other studies.^26^ It is, therefore, possible that the characteristics of the other study's data, rather than being healthy individuals or not, are responsible for the differences between CRC patients and healthy individuals and increase the accuracy of the diagnosis in a pretense. In addition, the diagnostic model obtained in Zeller's study showed AUC = 0.85 in the test data, which at first glance appears to be a high performance,^26^ but the reason for this value may be that only two patients with CRC were in Stage 0, and the others were in more advanced stages. In fact, in Zeller's CRC diagnostic model, when the false positive rate is 7.7%, the true positive rate is less than 60% even for Stage III/IV.^26^ On the other hand, our CRC diagnostic model has a true positive rate of more than 60% for CRC that had progressed to stage II or higher (Table 2).

Nevertheless, *Gemella morbillorum, Peptostreptococcus stomatis,* and *Parvimonas micra*, which ranked among the most important bacterial for diagnosis in our CRC diagnostic model (Figure 3), have also been identified as CRC‐associated bacteria in other reports^20^, ^27^, ^40^, ^41^ including Zeller's study,^26^ suggesting that these bacterial species are likely to be increased commonly in CRC patients, regardless of geographical localization, technical protocols, and ethnicity. However, since, there have been no reports to date that these bacteria are involved in the development of CRC, it is possible that some bacteria have increased or decreased as a result of developing CRC (Figure 3). In this regard, it is important to note that several known tumor‐promoting bacteria, such as *Fusobacterium nucleatum,* ^11^, ^12^, ^13^ *Peptostreptococcus anaerobius,* ^19^ *Porphyromonas gingivalis,* ^20^ and *Porphyromonas asaccharolytica,* ^20^ are not listed in Figure 3, as they overlap in appearance pattern with the above‐mentioned bacteria and do not further improve the diagnostic efficiency of CRC. Therefore, the bacteria shown in Figure 3, which are good diagnostic markers for CRC, may not necessarily be the bacteria involved in the development of CRC. In other words, we should be cautious about discussing the causal relationship between gut bacteria and CRC based only on quantitative changes in gut bacteria, and biological function analysis of gut bacteria is also necessary.

Although we did not perform FOBT in this cohort study, the test data of cohort‐3 included 21 clean healthy individuals and 13 healthy individuals with colorectal polyps who had false positive results on FOBT. Notably, 76.2% (95% confidence interval, 57.1%–90.5%) of these 21 clean healthy individuals and 76.9% (53.8%–100.0%) of these 13 healthy individuals with colorectal polyps tested negative in our CRC diagnostic model, suggesting that combining FOBT with our gut microbiota‐based diagnostic model may reduce false positives by more than 75%. Moreover, as mentioned earlier, the FOBT is known to have a poor positive rate for proximal colon cancer.^42^ On the other hand, our model showed no statistically significant difference in the positive rate in any part of the colorectum (Table 4). Proximal colon cancer is known to have a low survival rate, which may be partly due to the delay in detection caused by the low true positive rate by FOBT. Therefore, our model may also be useful for improving the survival rate of proximal colon cancer. However, contrary to our expectations, the CRC diagnostic model we developed could not sufficiently achieve early detection, which is an important issue in CRC screening tests. This indicates that in the early stages of CRC, the gut microbiota is not altered enough to be detected by stool examination, implying that there may be limitations in analyzing the gut microbiota in feces for the diagnosis of early stage CRC. Further studies are therefore needed to overcome this limitation.

## CONFLICT OF INTEREST

None.

## AUTHOR CONTRIBUTIONS

Eiji Hara and Satoshi Nagayama designed the experiments. Yusuke Konishi did most of the analyses. Shintaro Okumura collected and organized the clinical information of the healthy subjects and colorectal cancer patients who participated in this study. All the authors discussed the results and commented on the manuscript.

## ETHICS STATEMENT

This study was approved by the Institutional Review Board of Osaka University, JFCR hospital, Kyoto University Hospital, and Osaka City University Hospital.

## Supporting information


Figure S1
Figure S2.
Figure S3.
Figure S4.
Table S1.
Table S2.

**Appendix** **S1**: Supporting Information

## Data Availability

Microbiome analysis (bacterial 16S rRNA gene meta‐sequence) data generated in this study have been deposited in the DNA Data Bank of Japan (DDBJ) with the accession codes DRA011735 (cohort‐1) or DRA011736 (cohort‐2) or DRA012966 or DRA013152 (cohort‐3) (https://www.ddbj.nig.ac.jp). The deposited data are available in NCBI under accession numbers DRA011735, DRA011736, DRA012966, and DRA013152. The databases referred in microbiome analysis are as follows: SILVA (https://www.arb‐silva.de/) and National Center for Biotechnology Information (NCBI) (https://www.ncbi.nlm.nih.gov/).
