# Supplementary material for: Development and evaluation of a colorectal cancer screening method using machine learning‐based gut microbiota analysis
Source: Cancer Med. 2022 Mar 22;11(16):3194–206. doi: 10.1002/cam4.4671 (PMC9385600; doi:10.1002/cam4.4671)
Supplement: Supplementary file 1 — Figure S1 Figure S2. Figure S3. Figure S4. Table S1. Table S2. [file CAM4-11-3194-s002.pdf]

Figure S1

(A)

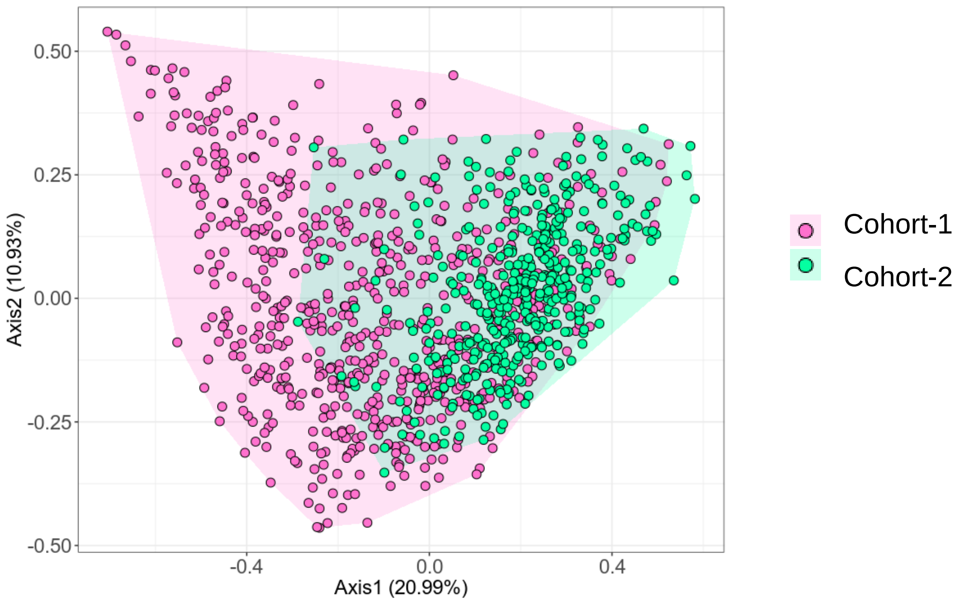

(B)

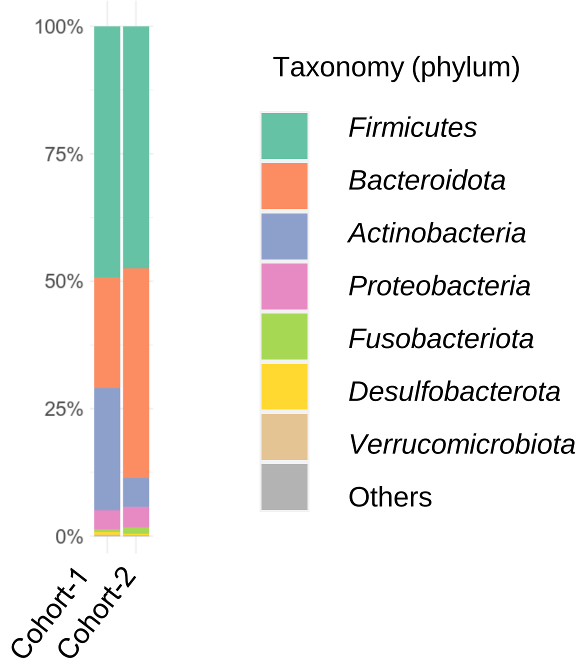

Figure S1. Differences in gut microbiota between cohort-1 and cohort-2 samples
(A) Principal co-ordinate analysis of the weighted UniFrac distance matrix.
(B) Gut microbiota composition of the two cohorts.

Figure S2

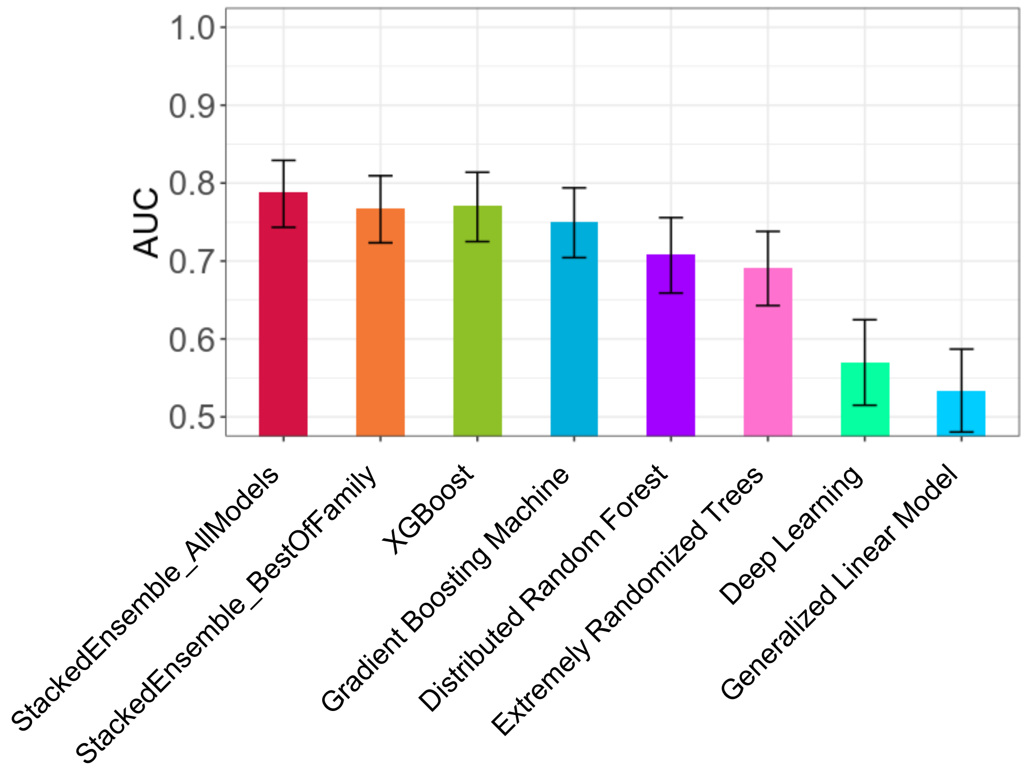

Figure S2. Performance comparison of seven machine-learning algorithms using cohort-1

Among the models created using each algorithm, the model with the highest AUC by cross-validation prediction was selected, and the AUC of these models for the test dataset was further compared. The training dataset consisted of 120 clean healthy individuals and 120 CRC patients, and the test dataset consisted of 150 clean healthy individuals and 133 CRC patients. Error bars indicate 95% confidence intervals, with 10,000 bootstrap replicates.

Figure S3

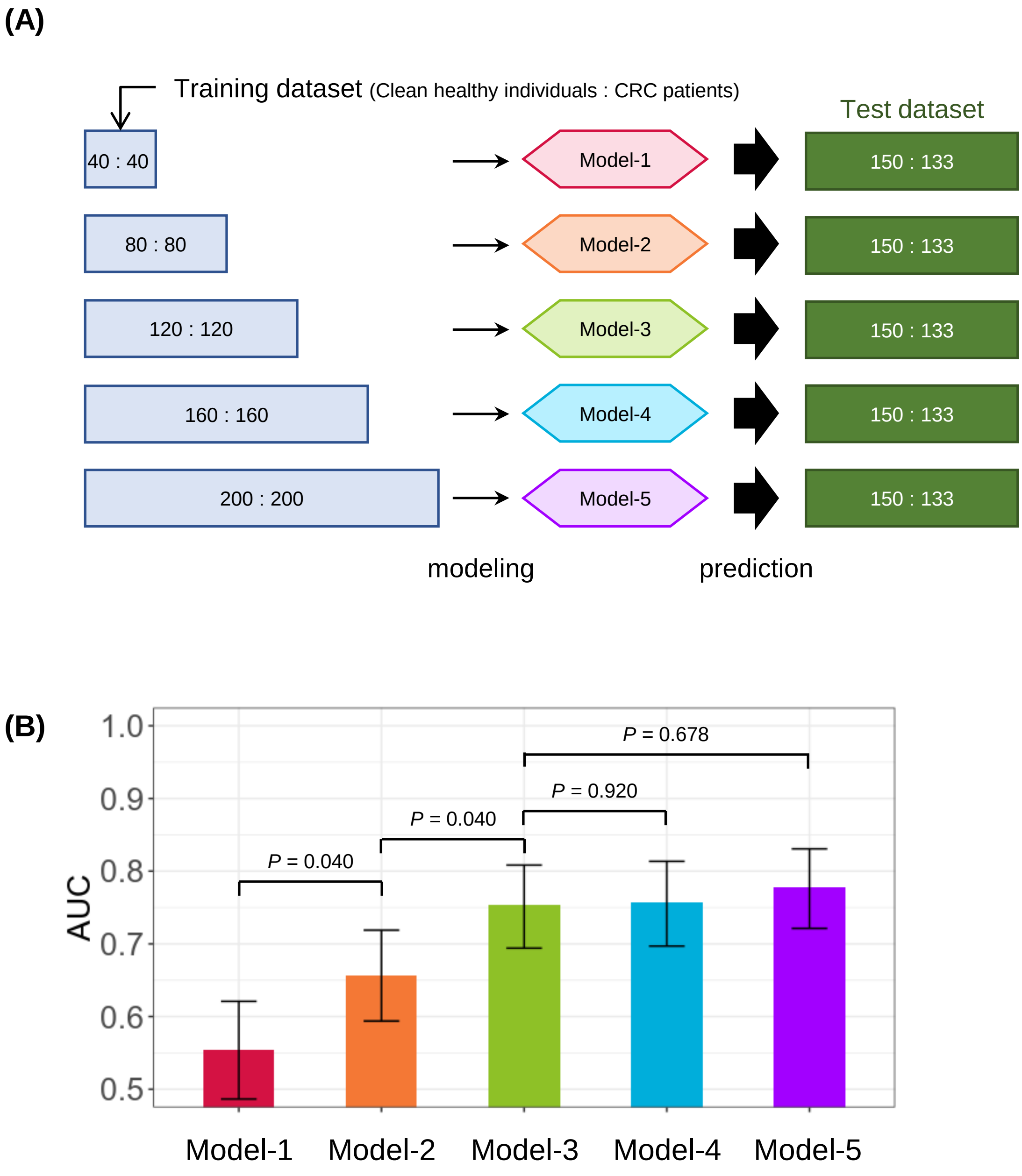

Figure S3. Determination of the sample size needed to create a CRC diagnostic model
(A) Flow diagram. We tried to create a CRC diagnostic model by incrementally increasing the number of clean healthy individuals and CRC patients in the cohort-1 training dataset to 40 each, 80 each, 120 each, 160 each, and 200 each. The test data set consists of 150 clean healthy individuals and 133 CRC patients and is exactly the same for all five cases.
(B) Comparison of the AUC for the test dataset shown by five models trained on different sized training datasets. Error bars indicate 95% confidence intervals with 10,000 bootstrap replicates. Statistical significance was determined with a bootstrapping method with 10,000 resamples. *P* values < 0.05 were considered significant.

Figure S4

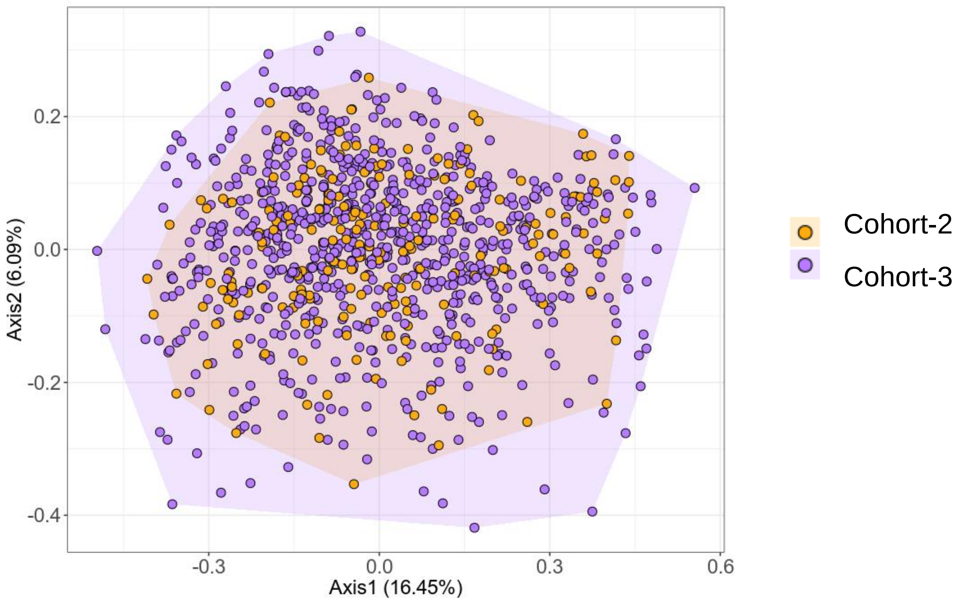

Figure S4. Differences in gut microbiota between cohort-2 and cohort-3 samples
Principal co-ordinate analysis of the weighted UniFrac distance matrix.

Table S1

Table S1. PCR primers used in this study

**Primers for 16S rRNA gene-sequencing**		
**1st PCR**		
**Primer name**		**Sequence (overhangs are underlined)**
16S-27Fmod	Forward	TCGTCGGCAGCGTCAGATGTGTATAAGAGACAGAGRGTTTGATYMTGGCTCAG
16S-338R	Reverse	GTCTCGTGGGCTCGGAGATGTGTATAAGAGACAGTGCTGCCTCCCGTAGGAGT
**2nd PCR**		
Nextera DNA Indexes (Illumina Inc., San Diego, CA, USA)		
**Primers for quantitative real-time PCR (Guo, et al., 2018)**		
**Primer name**		**Sequence**
Internal control (16S rRNA gene)	Forward	CGTCAGCTCGTGYCGTGAG
	Reverse	CGTCRTCCCCRCCTTCC
Fusobacterium nucleatum	Forward	TTCAATAAAAGTGGCAGGTCAAG
	Reverse	TAACAACACATGCAGGTCAATGG
Faecalibacterium prausnitzii	Forward	GGAGGATTGACCCCTTCAGT
	Reverse	CTGGTCCCGAAGAAACACAT
Bifidobacterium	Forward	TCGCGTCCGGTGTGAAAG
	Reverse	CCACATCCAGCATCCAC

Table S2

Table S2. Summary of the study participants

Study population	Clean HI	CRC patients Stage 0	I	II	III	IV
**Training data**						
Model-1	40	3	6	14	12	5
Model-2	80	4	16	26	23	11
Model-3	120	4	27	31	42	16
Model-4	160	7	39	42	54	18
Model-5	200	8	49	50	71	22
**Test data**	150	6	32	34	49	12

HI: Healthy individuals
